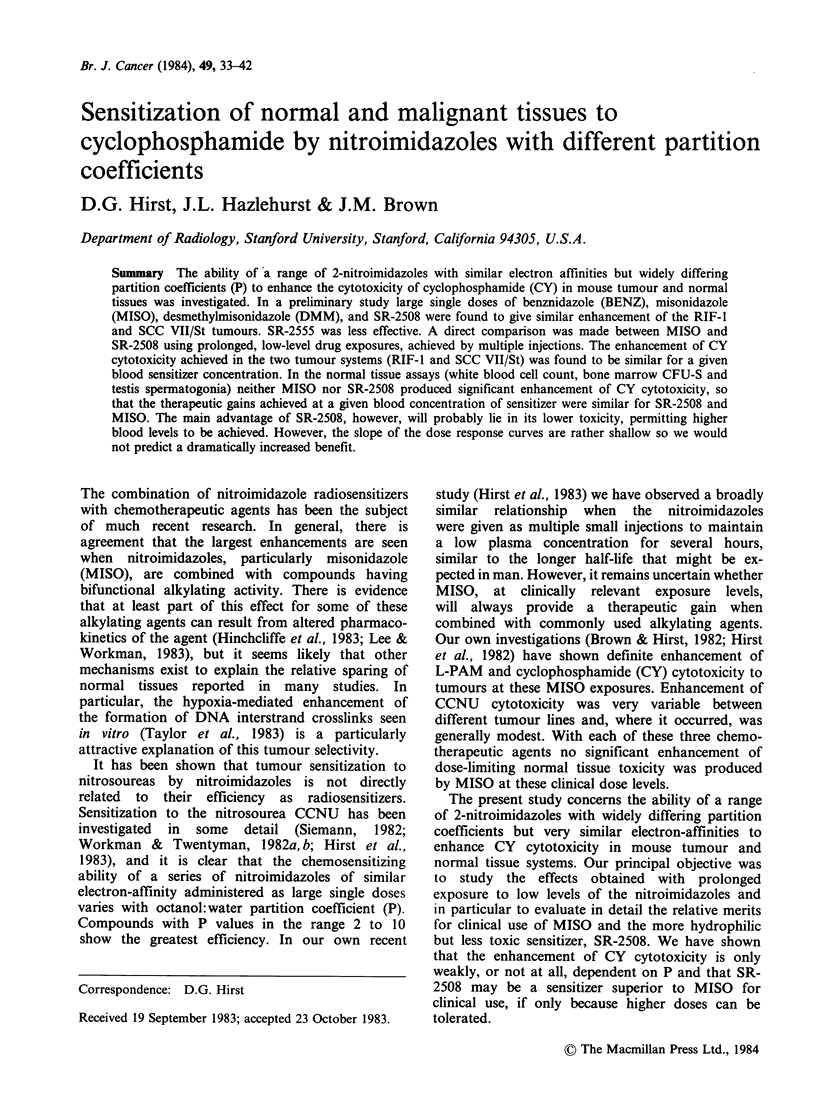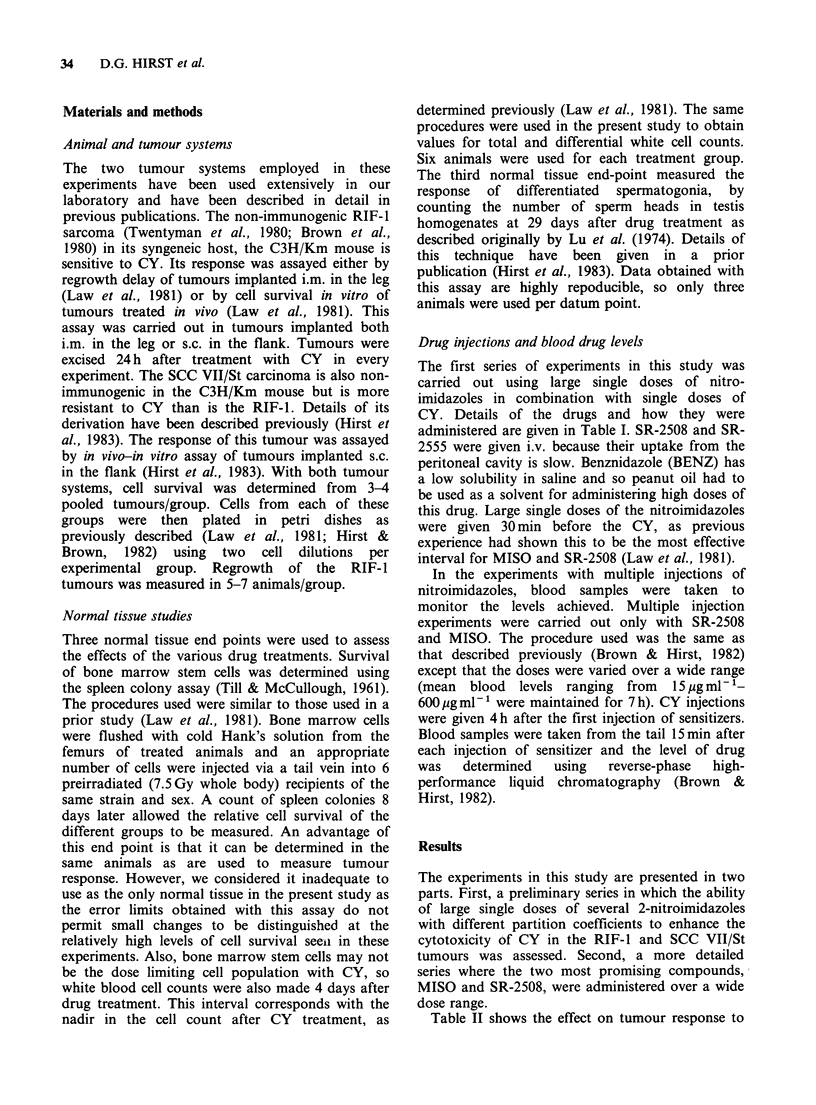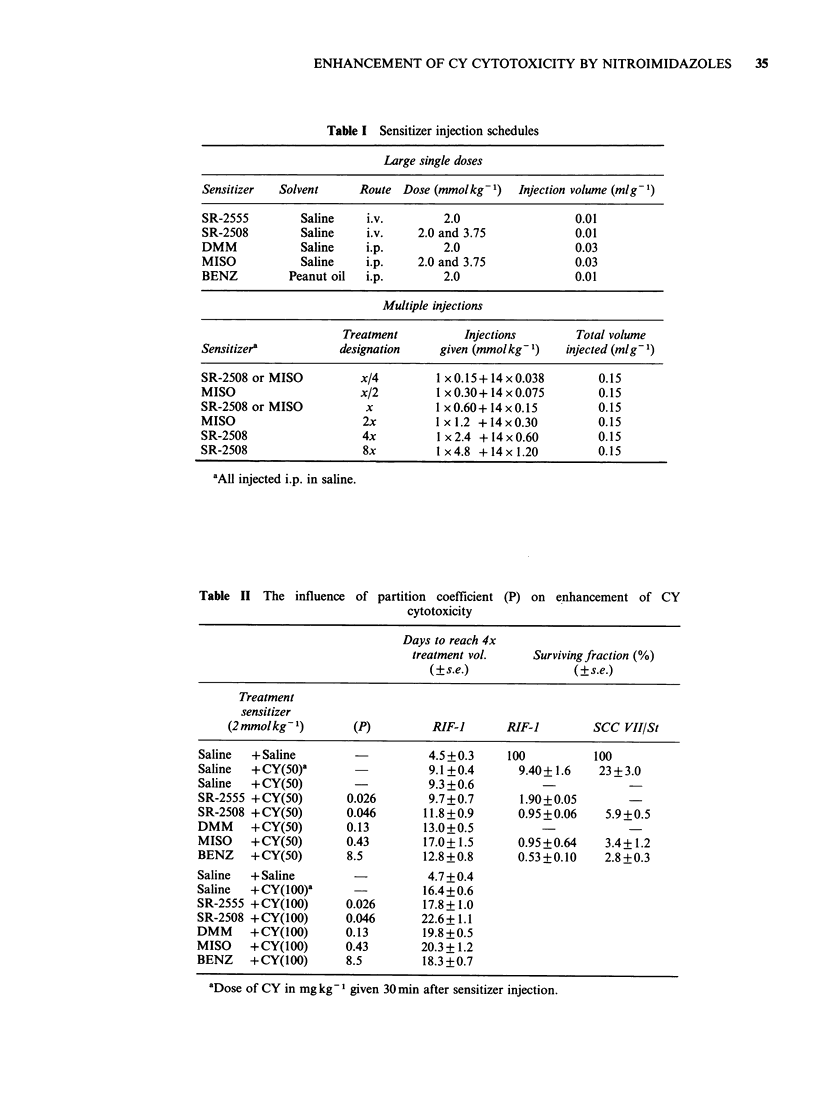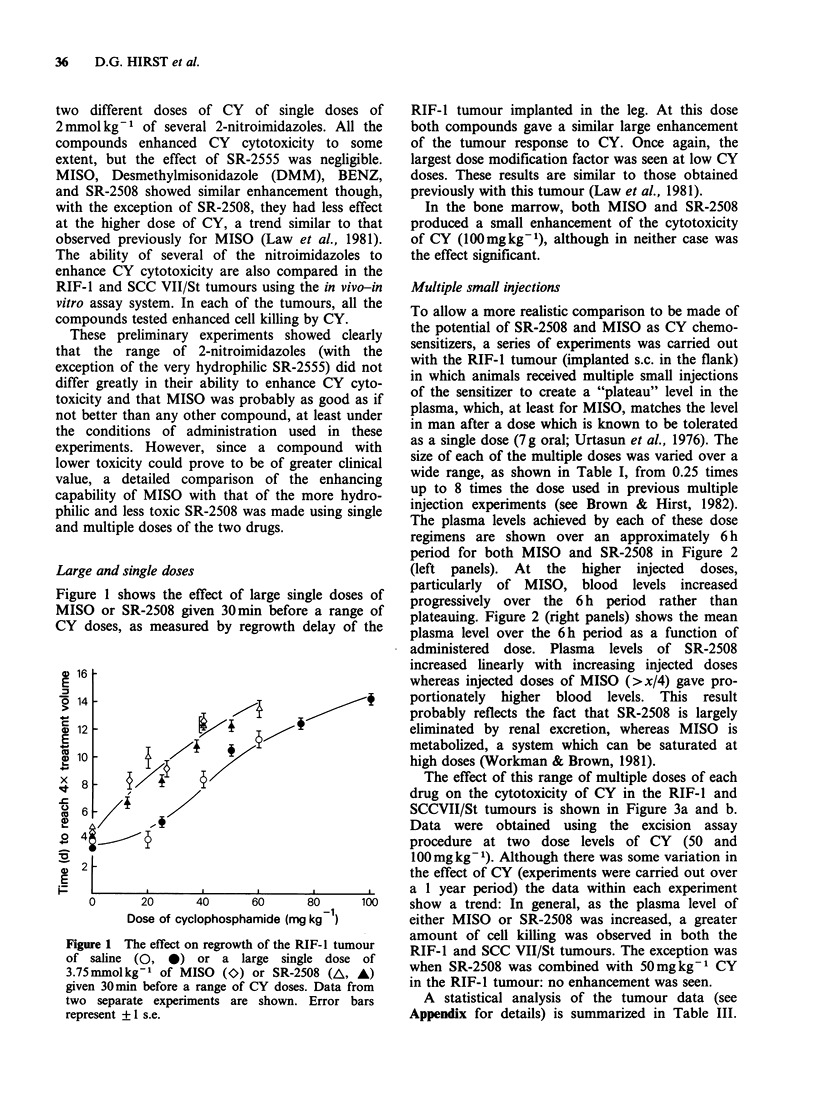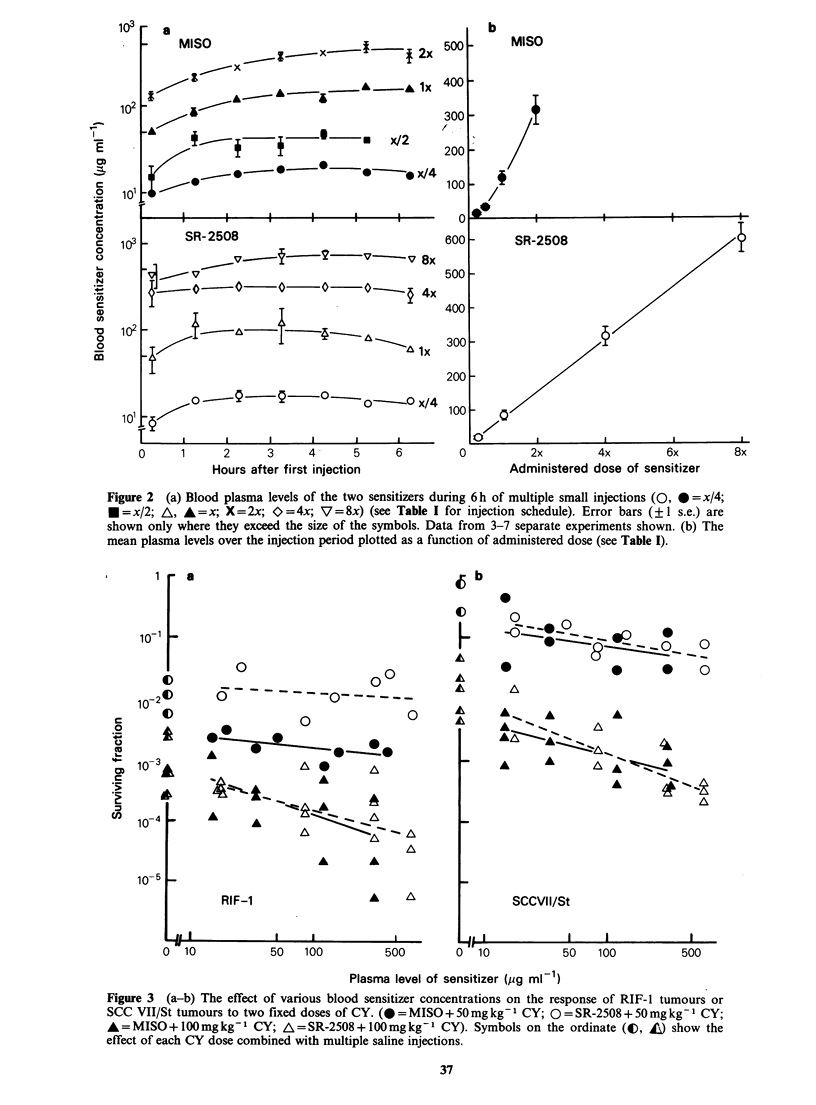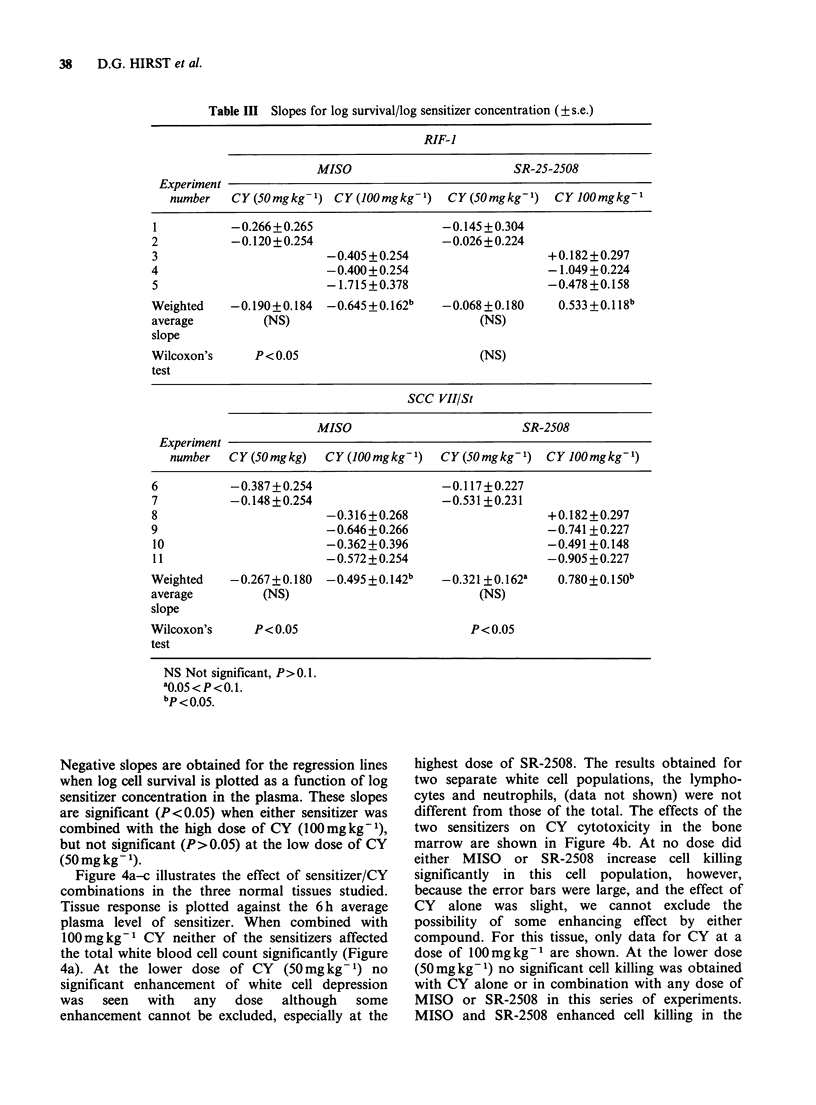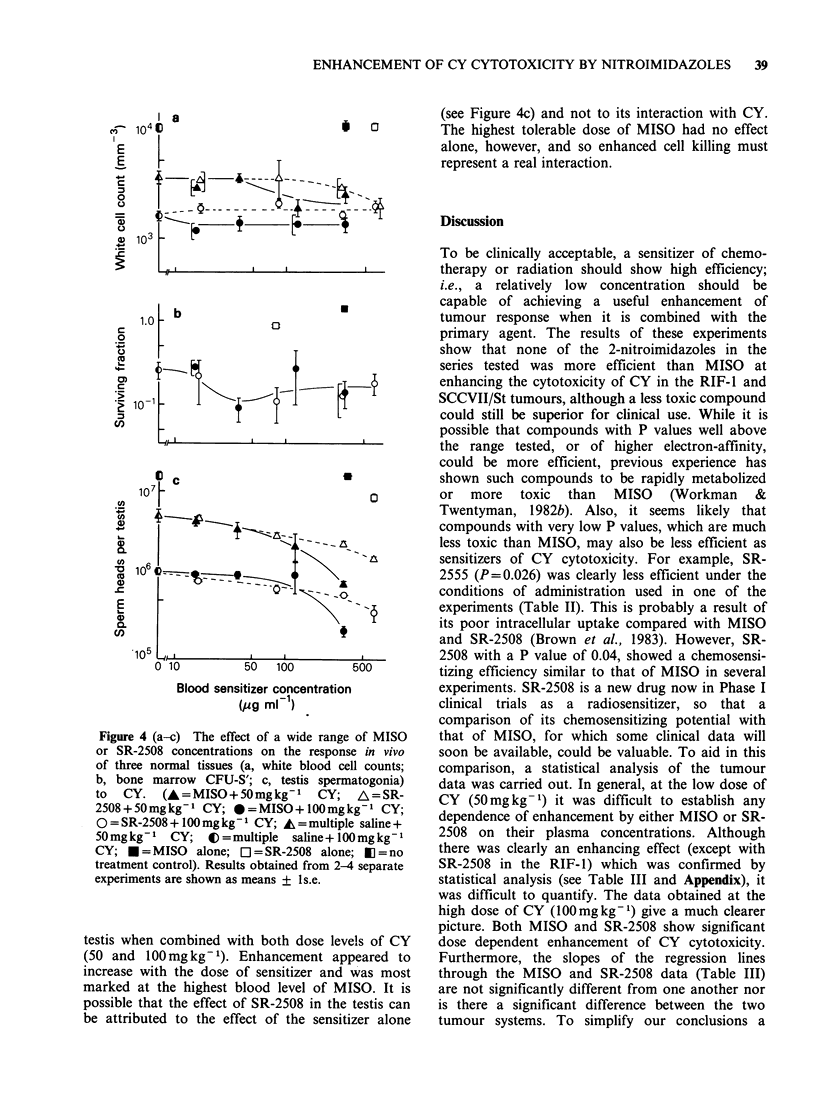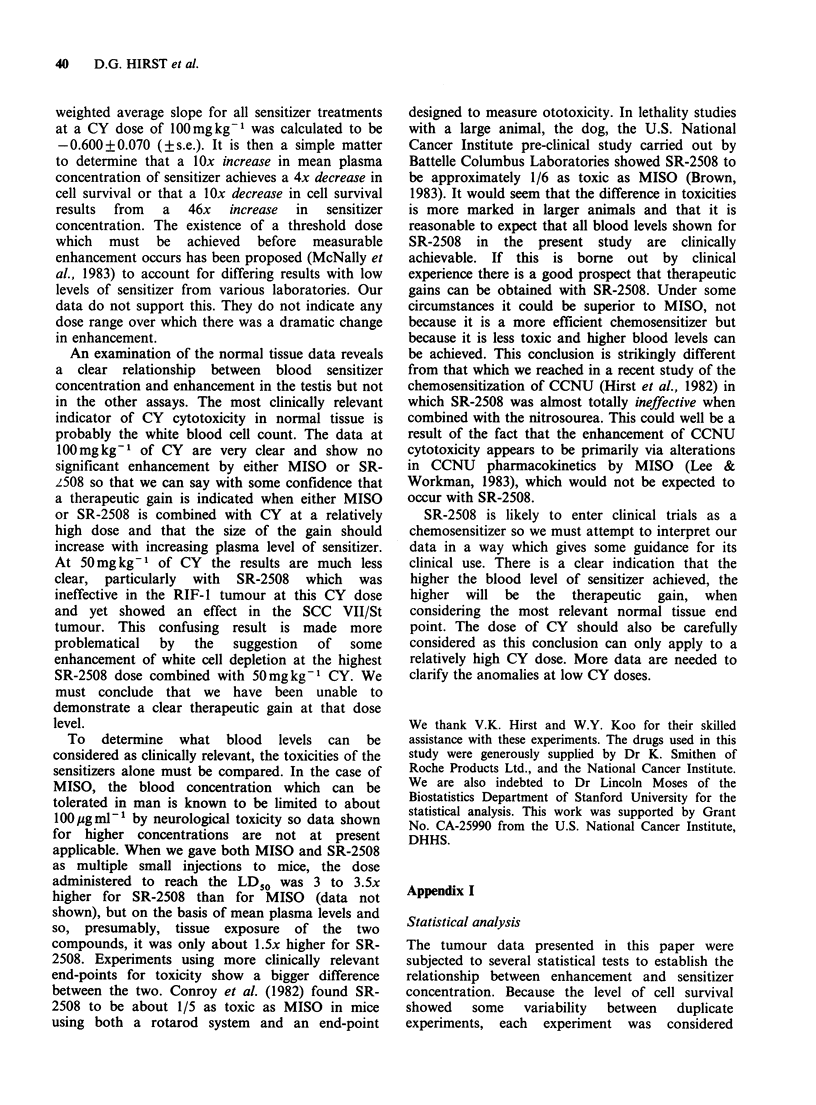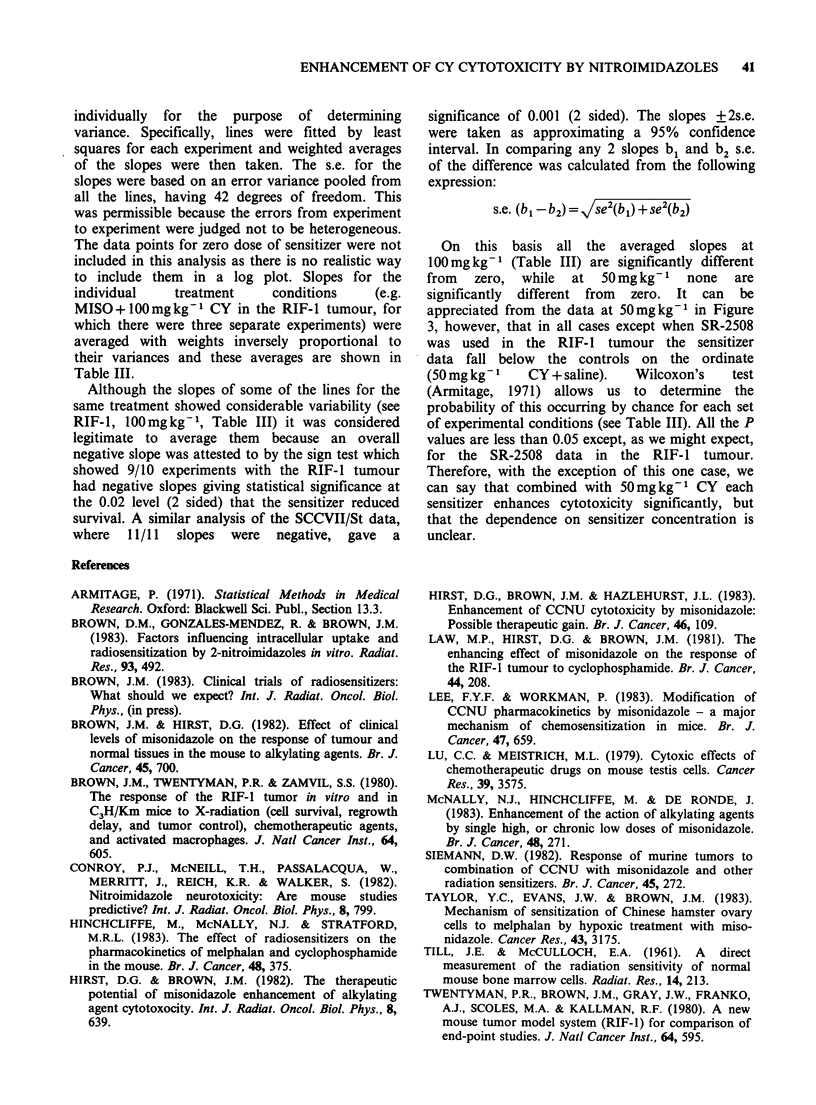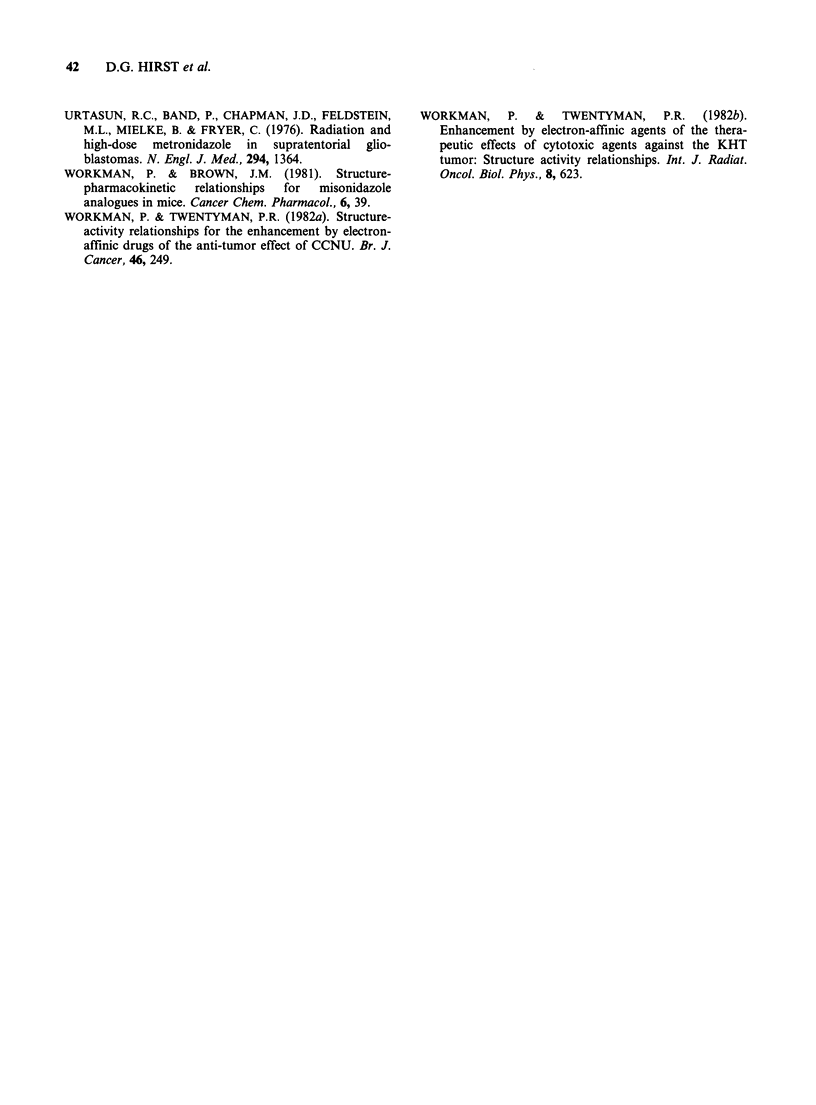# Sensitization of normal and malignant tissue to cyclophosphamide by nitroimidazoles with different partition coefficients.

**DOI:** 10.1038/bjc.1984.6

**Published:** 1984-01

**Authors:** D. G. Hirst, J. L. Hazlehurst, J. M. Brown

## Abstract

The ability of a range of 2-nitroimidazoles with similar electron affinities but widely differing partition coefficients (P) to enhance the cytotoxicity of cyclophosphamide (CY) in mouse tumour and normal tissues was investigated. In a preliminary study large single doses of benznidazole (BENZ), misonidazole (MISO), desmethylmisonidazole (DMM), and SR-2508 were found to give similar enhancement of the RIF-1 and SCC VII/St tumours. SR-2555 was less effective. A direct comparison was made between MISO and SR-2508 using prolonged, low-level drug exposures, achieved by multiple injections. The enhancement of CY cytotoxicity achieved in the two tumour systems (RIF-1 and SCC VII/St) was found to be similar for a given blood sensitizer concentration. In the normal tissue assays (white blood cell count, bone marrow CFU-S and testis spermatogonia) neither MISO nor SR-2508 produced significant enhancement of CY cytotoxicity, so that the therapeutic gains achieved at a given blood concentration of sensitizer were similar for SR-2508 and MISO. The main advantage of SR-2508, however, will probably lie in its lower toxicity, permitting higher blood levels to be achieved. However, the slope of the dose response curves are rather shallow so we would not predict a dramatically increased benefit.


*Br. J. Cancer* (1984), **49**, 33–42

### Sensitization of normal and malignant tissues to cyclophosphamide by nitroimidazoles with different partition coefficients

D.G. Hirst, J.L. Hazlehurst & J.M. Brown

*Department of Radiology, Stanford University, Stanford, California 94305, U.S.A.*

**Summary** The ability of a range of 2-nitroimidazoles with similar electron affinities but widely differing partition coefficients (P) to enhance the cytotoxicity of cyclophosphamide (CY) in mouse tumour and normal tissues was investigated. In a preliminary study large single doses of benznidazole (BENZ), misonidazole (MISO), desmethylmisonidazole (DMM), and SR-2508 were found to give similar enhancement of the RIF-1 and SCC VII/St tumours. SR-2555 was less effective. A direct comparison was made between MISO and SR-2508 using prolonged, low-level drug exposures, achieved by multiple injections. The enhancement of CY cytotoxicity achieved in the two tumour systems (RIF-1 and SCC VII/St) was found to be similar for a given blood sensitizer concentration. In the normal tissue assays (white blood cell count, bone marrow CFU-S and testis spermatogonia) neither MISO nor SR-2508 produced significant enhancement of CY cytotoxicity, so that the therapeutic gains achieved at a given blood concentration of sensitizer were similar for SR-2508 and MISO. The main advantage of SR-2508, however, will probably lie in its lower toxicity, permitting higher blood levels to be achieved. However, the slope of the dose response curves are rather shallow so we would not predict a dramatically increased benefit.

The combination of nitroimidazole radiosensitizers with chemotherapeutic agents has been the subject of much recent research. In general, there is agreement that the largest enhancements are seen when nitroimidazoles, particularly misonidazole (MISO), are combined with compounds having bifunctional alkylating activity. There is evidence that at least part of this effect for some of these alkylating agents can result from altered pharmacokinetics of the agent (Hinchcliffe *et al.*, 1983; Lee & Workman, 1983), but it seems likely that other mechanisms exist to explain the relative sparing of normal tissues reported in many studies. In particular, the hypoxia-mediated enhancement of the formation of DNA interstrand crosslinks seen *in vitro* (Taylor *et al.*, 1983) is a particularly attractive explanation of this tumour selectivity.

It has been shown that tumour sensitization to nitrosoureas by nitroimidazoles is not directly related to their efficiency as radiosensitizers. Sensitization to the nitrosourea CCNU has been investigated in some detail (Siemann, 1982; Workman & Twentyman, 1982*a,b*; Hirst *et al.*, 1983), and it is clear that the chemosensitizing ability of a series of nitroimidazoles of similar electron-affinity administered as large single doses varies with octanol:water partition coefficient (P). Compounds with P values in the range 2 to 10 show the greatest efficiency. In our own recent study (Hirst *et al.*, 1983) we have observed a broadly similar relationship when the nitroimidazoles were given as multiple small injections to maintain a low plasma concentration for several hours, similar to the longer half-life that might be expected in man. However, it remains uncertain whether MISO, at clinically relevant exposure levels, will always provide a therapeutic gain when combined with commonly used alkylating agents. Our own investigations (Brown & Hirst, 1982; Hirst *et al.*, 1982) have shown definite enhancement of L-PAM and cyclophosphamide (CY) cytotoxicity to tumours at these MISO exposures. Enhancement of CCNU cytotoxicity was very variable between different tumour lines and, where it occurred, was generally modest. With each of these three chemotherapeutic agents no significant enhancement of dose-limiting normal tissue toxicity was produced by MISO at these clinical dose levels.

The present study concerns the ability of a range of 2-nitroimidazoles with widely differing partition coefficients but very similar electron-affinities to enhance CY cytotoxicity in mouse tumour and normal tissue systems. Our principal objective was to study the effects obtained with prolonged exposure to low levels of the nitroimidazoles and in particular to evaluate in detail the relative merits for clinical use of MISO and the more hydrophilic but less toxic sensitizer, SR-2508. We have shown that the enhancement of CY cytotoxicity is only weakly, or not at all, dependent on P and that SR-2508 may be a sensitizer superior to MISO for clinical use, if only because higher doses can be tolerated.

Correspondence: D.G. Hirst

Received 19 September 1983; accepted 23 October 1983.

© The Macmillan Press Ltd., 1984

### Materials and methods

*Animal and tumour systems*

The two tumour systems employed in these experiments have been used extensively in our laboratory and have been described in detail in previous publications. The non-immunogenic RIF-1 sarcoma (Twentyman *et al.*, 1980; Brown *et al.*, 1980) in its syngeneic host, the C3H/Km mouse is sensitive to CY. Its response was assayed either by regrowth delay of tumours implanted i.m. in the leg (Law *et al.*, 1981) or by cell survival *in vitro* of tumours treated *in vivo* (Law *et al.*, 1981). This assay was carried out in tumours implanted both i.m. in the leg or s.c. in the flank. Tumours were excised 24 h after treatment with CY in every experiment. The SCC VII/St carcinoma is also non-immunogenic in the C3H/Km mouse but is more resistant to CY than is the RIF-1. Details of its derivation have been described previously (Hirst *et al.*, 1983). The response of this tumour was assayed by *in vivo–in vitro* assay of tumours implanted s.c. in the flank (Hirst *et al.*, 1983). With both tumour systems, cell survival was determined from 3–4 pooled tumours/group. Cells from each of these groups were then plated in petri dishes as previously described (Law *et al.*, 1981; Hirst & Brown, 1982) using two cell dilutions per experimental group. Regrowth of the RIF-1 tumours was measured in 5–7 animals/group.

*Normal tissue studies*

Three normal tissue end points were used to assess the effects of the various drug treatments. Survival of bone marrow stem cells was determined using the spleen colony assay (Till & McCullough, 1961). The procedures used were similar to those used in a prior study (Law *et al.*, 1981). Bone marrow cells were flushed with cold Hank's solution from the femurs of treated animals and an appropriate number of cells were injected via a tail vein into 6 preirradiated (7.5 Gy whole body) recipients of the same strain and sex. A count of spleen colonies 8 days later allowed the relative cell survival of the different groups to be measured. An advantage of this end point is that it can be determined in the same animals as are used to measure tumour response. However, we considered it inadequate to use as the only normal tissue in the present study as the error limits obtained with this assay do not permit small changes to be distinguished at the relatively high levels of cell survival seen in these experiments. Also, bone marrow stem cells may not be the dose limiting cell population with CY, so white blood cell counts were also made 4 days after drug treatment. This interval corresponds with the nadir in the cell count after CY treatment, as determined previously (Law *et al.*, 1981). The same procedures were used in the present study to obtain values for total and differential white cell counts. Six animals were used for each treatment group. The third normal tissue end-point measured the response of differentiated spermatogonia, by counting the number of sperm heads in testis homogenates at 29 days after drug treatment as described originally by Lu *et al.* (1974). Details of this technique have been given in a prior publication (Hirst *et al.*, 1983). Data obtained with this assay are highly repoducible, so only three animals were used per datum point.

*Drug injections and blood drug levels*

The first series of experiments in this study was carried out using large single doses of nitro-imidazoles in combination with single doses of CY. Details of the drugs and how they were administered are given in Table I. SR-2508 and SR-2555 were given i.v. because their uptake from the peritoneal cavity is slow. Benznidazole (BENZ) has a low solubility in saline and so peanut oil had to be used as a solvent for administering high doses of this drug. Large single doses of the nitroimidazoles were given 30 min before the CY, as previous experience had shown this to be the most effective interval for MISO and SR-2508 (Law *et al.*, 1981).

In the experiments with multiple injections of nitroimidazoles, blood samples were taken to monitor the levels achieved. Multiple injection experiments were carried out only with SR-2508 and MISO. The procedure used was the same as that described previously (Brown & Hirst, 1982) except that the doses were varied over a wide range (mean blood levels ranging from $15\,\mu g\,ml^{-1}$–$600\,\mu g\,ml^{-1}$ were maintained for 7 h). CY injections were given 4 h after the first injection of sensitizers. Blood samples were taken from the tail 15 min after each injection of sensitizer and the level of drug was determined using reverse-phase high-performance liquid chromatography (Brown & Hirst, 1982).

### Results

The experiments in this study are presented in two parts. First, a preliminary series in which the ability of large single doses of several 2-nitroimidazoles with different partition coefficients to enhance the cytotoxicity of CY in the RIF-1 and SCC VII/St tumours was assessed. Second, a more detailed series where the two most promising compounds, MISO and SR-2508, were administered over a wide dose range.

Table II shows the effect on tumour response to

**Table I** Sensitizer injection schedules

*Large single doses*

| *Sensitizer* | *Solvent* | *Route* | *Dose* (*mmol kg*$^{-1}$) | *Injection volume* (*ml g*$^{-1}$) |
|---|---|---|---|---|
| SR-2555 | Saline | i.v. | 2.0 | 0.01 |
| SR-2508 | Saline | i.v. | 2.0 and 3.75 | 0.01 |
| DMM | Saline | i.p. | 2.0 | 0.03 |
| MISO | Saline | i.p. | 2.0 and 3.75 | 0.03 |
| BENZ | Peanut oil | i.p. | 2.0 | 0.01 |

*Multiple injections*

| *Sensitizer*[a] | *Treatment designation* | *Injections given* (*mmol kg*$^{-1}$) | *Total volume injected* (*ml g*$^{-1}$) |
|---|---|---|---|
| SR-2508 or MISO | $x/4$ | $1\times0.15+14\times0.038$ | 0.15 |
| MISO | $x/2$ | $1\times0.30+14\times0.075$ | 0.15 |
| SR-2508 or MISO | $x$ | $1\times0.60+14\times0.15$ | 0.15 |
| MISO | $2x$ | $1\times1.2+14\times0.30$ | 0.15 |
| SR-2508 | $4x$ | $1\times2.4+14\times0.60$ | 0.15 |
| SR-2508 | $8x$ | $1\times4.8+14\times1.20$ | 0.15 |

[a]All injected i.p. in saline.

**Table II** The influence of partition coefficient (P) on enhancement of CY cytotoxicity

| *Treatment sensitizer* (*2 mmol kg*$^{-1}$) | (*P*) | *Days to reach 4x treatment vol.* ($\pm$*s.e.*) *RIF-1* | *Surviving fraction* (%) ($\pm$*s.e.*) *RIF-1* | *Surviving fraction* (%) ($\pm$*s.e.*) *SCC VII/St* |
|---|---|---|---|---|
| Saline +Saline | — | 4.5±0.3 | 100 | 100 |
| Saline +CY(50)[a] | — | 9.1±0.4 | 9.40±1.6 | 23±3.0 |
| Saline +CY(50) | — | 9.3±0.6 | — | — |
| SR-2555 +CY(50) | 0.026 | 9.7±0.7 | 1.90±0.05 | — |
| SR-2508 +CY(50) | 0.046 | 11.8±0.9 | 0.95±0.06 | 5.9±0.5 |
| DMM +CY(50) | 0.13 | 13.0±0.5 | — | — |
| MISO +CY(50) | 0.43 | 17.0±1.5 | 0.95±0.64 | 3.4±1.2 |
| BENZ +CY(50) | 8.5 | 12.8±0.8 | 0.53±0.10 | 2.8±0.3 |
| Saline +Saline | — | 4.7±0.4 | | |
| Saline +CY(100)[a] | — | 16.4±0.6 | | |
| SR-2555 +CY(100) | 0.026 | 17.8±1.0 | | |
| SR-2508 +CY(100) | 0.046 | 22.6±1.1 | | |
| DMM +CY(100) | 0.13 | 19.8±0.5 | | |
| MISO +CY(100) | 0.43 | 20.3±1.2 | | |
| BENZ +CY(100) | 8.5 | 18.3±0.7 | | |

[a]Dose of CY in mg kg$^{-1}$ given 30 min after sensitizer injection.

two different doses of CY of single doses of 2mmol kg$^{-1}$ of several 2-nitroimidazoles. All the compounds enhanced CY cytotoxicity to some extent, but the effect of SR-2555 was negligible. MISO, Desmethylmisonidazole (DMM), BENZ, and SR-2508 showed similar enhancement though, with the exception of SR-2508, they had less effect at the higher dose of CY, a trend similar to that observed previously for MISO (Law *et al.*, 1981). The ability of several of the nitroimidazoles to enhance CY cytotoxicity are also compared in the RIF-1 and SCC VII/St tumours using the *in vivo–in vitro* assay system. In each of the tumours, all the compounds tested enhanced cell killing by CY.

These preliminary experiments showed clearly that the range of 2-nitroimidazoles (with the exception of the very hydrophilic SR-2555) did not differ greatly in their ability to enhance CY cytotoxicity and that MISO was probably as good as if not better than any other compound, at least under the conditions of administration used in these experiments. However, since a compound with lower toxicity could prove to be of greater clinical value, a detailed comparison of the enhancing capability of MISO with that of the more hydrophilic and less toxic SR-2508 was made using single and multiple doses of the two drugs.

*Large and single doses*

Figure 1 shows the effect of large single doses of MISO or SR-2508 given 30 min before a range of CY doses, as measured by regrowth delay of the RIF-1 tumour implanted in the leg. At this dose both compounds gave a similar large enhancement of the tumour response to CY. Once again, the largest dose modification factor was seen at low CY doses. These results are similar to those obtained previously with this tumour (Law *et al.*, 1981).

In the bone marrow, both MISO and SR-2508 produced a small enhancement of the cytotoxicity of CY (100 mg kg$^{-1}$), although in neither case was the effect significant.

**Figure 1** The effect on regrowth of the RIF-1 tumour of saline (○, ●) or a large single dose of 3.75 mmol kg$^{-1}$ of MISO (◇) or SR-2508 (△, ▲) given 30 min before a range of CY doses. Data from two separate experiments are shown. Error bars represent ±1 s.e.

*Multiple small injections*

To allow a more realistic comparison to be made of the potential of SR-2508 and MISO as CY chemosensitizers, a series of experiments was carried out with the RIF-1 tumour (implanted s.c. in the flank) in which animals received multiple small injections of the sensitizer to create a "plateau" level in the plasma, which, at least for MISO, matches the level in man after a dose which is known to be tolerated as a single dose (7 g oral; Urtasun *et al.*, 1976). The size of each of the multiple doses was varied over a wide range, as shown in Table I, from 0.25 times up to 8 times the dose used in previous multiple injection experiments (see Brown & Hirst, 1982). The plasma levels achieved by each of these dose regimens are shown over an approximately 6 h period for both MISO and SR-2508 in Figure 2 (left panels). At the higher injected doses, particularly of MISO, blood levels increased progressively over the 6 h period rather than plateauing. Figure 2 (right panels) shows the mean plasma level over the 6 h period as a function of administered dose. Plasma levels of SR-2508 increased linearly with increasing injected doses whereas injected doses of MISO ($>x/4$) gave proportionately higher blood levels. This result probably reflects the fact that SR-2508 is largely eliminated by renal excretion, whereas MISO is metabolized, a system which can be saturated at high doses (Workman & Brown, 1981).

The effect of this range of multiple doses of each drug on the cytotoxicity of CY in the RIF-1 and SCCVII/St tumours is shown in Figure 3a and b. Data were obtained using the excision assay procedure at two dose levels of CY (50 and 100 mg kg$^{-1}$). Although there was some variation in the effect of CY (experiments were carried out over a 1 year period) the data within each experiment show a trend: In general, as the plasma level of either MISO or SR-2508 was increased, a greater amount of cell killing was observed in both the RIF-1 and SCC VII/St tumours. The exception was when SR-2508 was combined with 50 mg kg$^{-1}$ CY in the RIF-1 tumour: no enhancement was seen.

A statistical analysis of the tumour data (see **Appendix** for details) is summarized in Table III.

**Figure 2** (a) Blood plasma levels of the two sensitizers during 6 h of multiple small injections (○, ● = $x/4$; ■ = $x/2$; △, ▲ = $x$; **X** = $2x$; ◇ = $4x$; ▽ = $8x$) (see **Table I** for injection schedule). Error bars (±1 s.e.) are shown only where they exceed the size of the symbols. Data from 3–7 separate experiments shown. (b) The mean plasma levels over the injection period plotted as a function of administered dose (see **Table I**).

**Figure 3** (a–b) The effect of various blood sensitizer concentrations on the response of RIF-1 tumours or SCC VII/St tumours to two fixed doses of CY. (● = MISO + 50 mg kg⁻¹ CY; ○ = SR-2508 + 50 mg kg⁻¹ CY; ▲ = MISO + 100 mg kg⁻¹ CY; △ = SR-2508 + 100 mg kg⁻¹ CY). Symbols on the ordinate (◐, ◭) show the effect of each CY dose combined with multiple saline injections.

**Table III** Slopes for log survival/log sensitizer concentration (±s.e.)

| Experiment number | RIF-1 MISO CY (50 mg kg$^{-1}$) | RIF-1 MISO CY (100 mg kg$^{-1}$) | RIF-1 SR-25-2508 CY (50 mg kg$^{-1}$) | RIF-1 SR-25-2508 CY 100 mg kg$^{-1}$ |
|---|---|---|---|---|
| 1 | −0.266±0.265 | | −0.145±0.304 | |
| 2 | −0.120±0.254 | | −0.026±0.224 | |
| 3 | | −0.405±0.254 | | +0.182±0.297 |
| 4 | | −0.400±0.254 | | −1.049±0.224 |
| 5 | | −1.715±0.378 | | −0.478±0.158 |
| Weighted average slope | −0.190±0.184 (NS) | −0.645±0.162[b] | −0.068±0.180 (NS) | 0.533±0.118[b] |
| Wilcoxon's test | $P<0.05$ | | (NS) | |

| Experiment number | SCC VII/St MISO CY (50 mg kg) | SCC VII/St MISO CY (100 mg kg$^{-1}$) | SCC VII/St SR-2508 CY (50 mg kg$^{-1}$) | SCC VII/St SR-2508 CY 100 mg kg$^{-1}$) |
|---|---|---|---|---|
| 6 | −0.387±0.254 | | −0.117±0.227 | |
| 7 | −0.148±0.254 | | −0.531±0.231 | |
| 8 | | −0.316±0.268 | | +0.182±0.297 |
| 9 | | −0.646±0.266 | | −0.741±0.227 |
| 10 | | −0.362±0.396 | | −0.491±0.148 |
| 11 | | −0.572±0.254 | | −0.905±0.227 |
| Weighted average slope | −0.267±0.180 (NS) | −0.495±0.142[b] | −0.321±0.162[a] (NS) | 0.780±0.150[b] |
| Wilcoxon's test | $P<0.05$ | | $P<0.05$ | |

NS Not significant, $P>0.1$.

[a]$0.05<P<0.1$.

[b]$P<0.05$.

Negative slopes are obtained for the regression lines when log cell survival is plotted as a function of log sensitizer concentration in the plasma. These slopes are significant ($P<0.05$) when either sensitizer was combined with the high dose of CY (100 mg kg$^{-1}$), but not significant ($P>0.05$) at the low dose of CY (50 mg kg$^{-1}$).

Figure 4a–c illustrates the effect of sensitizer/CY combinations in the three normal tissues studied. Tissue response is plotted against the 6 h average plasma level of sensitizer. When combined with 100 mg kg$^{-1}$ CY neither of the sensitizers affected the total white blood cell count significantly (Figure 4a). At the lower dose of CY (50 mg kg$^{-1}$) no significant enhancement of white cell depression was seen with any dose although some enhancement cannot be excluded, especially at the highest dose of SR-2508. The results obtained for two separate white cell populations, the lymphocytes and neutrophils, (data not shown) were not different from those of the total. The effects of the two sensitizers on CY cytotoxicity in the bone marrow are shown in Figure 4b. At no dose did either MISO or SR-2508 increase cell killing significantly in this cell population, however, because the error bars were large, and the effect of CY alone was slight, we cannot exclude the possibility of some enhancing effect by either compound. For this tissue, only data for CY at a dose of 100 mg kg$^{-1}$ are shown. At the lower dose (50 mg kg$^{-1}$) no significant cell killing was obtained with CY alone or in combination with any dose of MISO or SR-2508 in this series of experiments. MISO and SR-2508 enhanced cell killing in the

**Figure 4** (a–c) The effect of a wide range of MISO or SR-2508 concentrations on the response *in vivo* of three normal tissues (a, white blood cell counts; b, bone marrow CFU-S′; c, testis spermatogonia) to CY. (▲=MISO+50 mg kg$^{-1}$ CY; △=SR-2508+50 mg kg$^{-1}$ CY; ●=MISO+100 mg kg$^{-1}$ CY; ○=SR-2508+100 mg kg$^{-1}$ CY; ◭=multiple saline+50 mg kg$^{-1}$ CY; ◐=multiple saline+100 mg kg$^{-1}$ CY; ■=MISO alone; □=SR-2508 alone; ◧=no treatment control). Results obtained from 2–4 separate experiments are shown as means ± 1s.e.

testis when combined with both dose levels of CY (50 and 100 mg kg$^{-1}$). Enhancement appeared to increase with the dose of sensitizer and was most marked at the highest blood level of MISO. It is possible that the effect of SR-2508 in the testis can be attributed to the effect of the sensitizer alone (see Figure 4c) and not to its interaction with CY. The highest tolerable dose of MISO had no effect alone, however, and so enhanced cell killing must represent a real interaction.

### Discussion

To be clinically acceptable, a sensitizer of chemotherapy or radiation should show high efficiency; *i.e.*, a relatively low concentration should be capable of achieving a useful enhancement of tumour response when it is combined with the primary agent. The results of these experiments show that none of the 2-nitroimidazoles in the series tested was more efficient than MISO at enhancing the cytotoxicity of CY in the RIF-1 and SCCVII/St tumours, although a less toxic compound could still be superior for clinical use. While it is possible that compounds with P values well above the range tested, or of higher electron-affinity, could be more efficient, previous experience has shown such compounds to be rapidly metabolized or more toxic than MISO (Workman & Twentyman, 1982*b*). Also, it seems likely that compounds with very low P values, which are much less toxic than MISO, may also be less efficient as sensitizers of CY cytotoxicity. For example, SR-2555 ($P=0.026$) was clearly less efficient under the conditions of administration used in one of the experiments (Table II). This is probably a result of its poor intracellular uptake compared with MISO and SR-2508 (Brown *et al.*, 1983). However, SR-2508 with a P value of 0.04, showed a chemosensitizing efficiency similar to that of MISO in several experiments. SR-2508 is a new drug now in Phase I clinical trials as a radiosensitizer, so that a comparison of its chemosensitizing potential with that of MISO, for which some clinical data will soon be available, could be valuable. To aid in this comparison, a statistical analysis of the tumour data was carried out. In general, at the low dose of CY (50 mg kg$^{-1}$) it was difficult to establish any dependence of enhancement by either MISO or SR-2508 on their plasma concentrations. Although there was clearly an enhancing effect (except with SR-2508 in the RIF-1) which was confirmed by statistical analysis (see Table III and **Appendix**), it was difficult to quantify. The data obtained at the high dose of CY (100 mg kg$^{-1}$) give a much clearer picture. Both MISO and SR-2508 show significant dose dependent enhancement of CY cytotoxicity. Furthermore, the slopes of the regression lines through the MISO and SR-2508 data (Table III) are not significantly different from one another nor is there a significant difference between the two tumour systems. To simplify our conclusions a weighted average slope for all sensitizer treatments at a CY dose of $100\,mg\,kg^{-1}$ was calculated to be $-0.600 \pm 0.070$ ($\pm$s.e.). It is then a simple matter to determine that a 10*x* *increase* in mean plasma concentration of sensitizer achieves a 4*x* *decrease* in cell survival or that a 10*x* *decrease* in cell survival results from a 46*x* *increase* in sensitizer concentration. The existence of a threshold dose which must be achieved before measurable enhancement occurs has been proposed (McNally *et al.*, 1983) to account for differing results with low levels of sensitizer from various laboratories. Our data do not support this. They do not indicate any dose range over which there was a dramatic change in enhancement.

An examination of the normal tissue data reveals a clear relationship between blood sensitizer concentration and enhancement in the testis but not in the other assays. The most clinically relevant indicator of CY cytotoxicity in normal tissue is probably the white blood cell count. The data at $100\,mg\,kg^{-1}$ of CY are very clear and show no significant enhancement by either MISO or SR-2508 so that we can say with some confidence that a therapeutic gain is indicated when either MISO or SR-2508 is combined with CY at a relatively high dose and that the size of the gain should increase with increasing plasma level of sensitizer. At $50\,mg\,kg^{-1}$ of CY the results are much less clear, particularly with SR-2508 which was ineffective in the RIF-1 tumour at this CY dose and yet showed an effect in the SCC VII/St tumour. This confusing result is made more problematical by the suggestion of some enhancement of white cell depletion at the highest SR-2508 dose combined with $50\,mg\,kg^{-1}$ CY. We must conclude that we have been unable to demonstrate a clear therapeutic gain at that dose level.

To determine what blood levels can be considered as clinically relevant, the toxicities of the sensitizers alone must be compared. In the case of MISO, the blood concentration which can be tolerated in man is known to be limited to about $100\,\mu g\,ml^{-1}$ by neurological toxicity so data shown for higher concentrations are not at present applicable. When we gave both MISO and SR-2508 as multiple small injections to mice, the dose administered to reach the $LD_{50}$ was 3 to 3.5*x* higher for SR-2508 than for MISO (data not shown), but on the basis of mean plasma levels and so, presumably, tissue exposure of the two compounds, it was only about 1.5*x* higher for SR-2508. Experiments using more clinically relevant end-points for toxicity show a bigger difference between the two. Conroy *et al.* (1982) found SR-2508 to be about 1/5 as toxic as MISO in mice using both a rotarod system and an end-point designed to measure ototoxicity. In lethality studies with a large animal, the dog, the U.S. National Cancer Institute pre-clinical study carried out by Battelle Columbus Laboratories showed SR-2508 to be approximately 1/6 as toxic as MISO (Brown, 1983). It would seem that the difference in toxicities is more marked in larger animals and that it is reasonable to expect that all blood levels shown for SR-2508 in the present study are clinically achievable. If this is borne out by clinical experience there is a good prospect that therapeutic gains can be obtained with SR-2508. Under some circumstances it could be superior to MISO, not because it is a more efficient chemosensitizer but because it is less toxic and higher blood levels can be achieved. This conclusion is strikingly different from that which we reached in a recent study of the chemosensitization of CCNU (Hirst *et al.*, 1982) in which SR-2508 was almost totally *ineffective* when combined with the nitrosourea. This could well be a result of the fact that the enhancement of CCNU cytotoxicity appears to be primarily via alterations in CCNU pharmacokinetics by MISO (Lee & Workman, 1983), which would not be expected to occur with SR-2508.

SR-2508 is likely to enter clinical trials as a chemosensitizer so we must attempt to interpret our data in a way which gives some guidance for its clinical use. There is a clear indication that the higher the blood level of sensitizer achieved, the higher will be the therapeutic gain, when considering the most relevant normal tissue end point. The dose of CY should also be carefully considered as this conclusion can only apply to a relatively high CY dose. More data are needed to clarify the anomalies at low CY doses.

We thank V.K. Hirst and W.Y. Koo for their skilled assistance with these experiments. The drugs used in this study were generously supplied by Dr K. Smithen of Roche Products Ltd., and the National Cancer Institute. We are also indebted to Dr Lincoln Moses of the Biostatistics Department of Stanford University for the statistical analysis. This work was supported by Grant No. CA-25990 from the U.S. National Cancer Institute, DHHS.

### Appendix I

### *Statistical analysis*

The tumour data presented in this paper were subjected to several statistical tests to establish the relationship between enhancement and sensitizer concentration. Because the level of cell survival showed some variability between duplicate experiments, each experiment was considered individually for the purpose of determining variance. Specifically, lines were fitted by least squares for each experiment and weighted averages of the slopes were then taken. The s.e. for the slopes were based on an error variance pooled from all the lines, having 42 degrees of freedom. This was permissible because the errors from experiment to experiment were judged not to be heterogeneous. The data points for zero dose of sensitizer were not included in this analysis as there is no realistic way to include them in a log plot. Slopes for the individual treatment conditions (e.g. MISO + 100 mg kg$^{-1}$ CY in the RIF-1 tumour, for which there were three separate experiments) were averaged with weights inversely proportional to their variances and these averages are shown in Table III.

Although the slopes of some of the lines for the same treatment showed considerable variability (see RIF-1, 100 mg kg$^{-1}$, Table III) it was considered legitimate to average them because an overall negative slope was attested to by the sign test which showed 9/10 experiments with the RIF-1 tumour had negative slopes giving statistical significance at the 0.02 level (2 sided) that the sensitizer reduced survival. A similar analysis of the SCCVII/St data, where 11/11 slopes were negative, gave a significance of 0.001 (2 sided). The slopes $\pm$2s.e. were taken as approximating a 95% confidence interval. In comparing any 2 slopes $b_1$ and $b_2$ s.e. of the difference was calculated from the following expression:

$$\text{s.e.}\ (b_1 - b_2) = \sqrt{se^2(b_1) + se^2(b_2)}$$

On this basis all the averaged slopes at 100 mg kg$^{-1}$ (Table III) are significantly different from zero, while at 50 mg kg$^{-1}$ none are significantly different from zero. It can be appreciated from the data at 50 mg kg$^{-1}$ in Figure 3, however, that in all cases except when SR-2508 was used in the RIF-1 tumour the sensitizer data fall below the controls on the ordinate (50 mg kg$^{-1}$ CY + saline). Wilcoxon's test (Armitage, 1971) allows us to determine the probability of this occurring by chance for each set of experimental conditions (see Table III). All the *P* values are less than 0.05 except, as we might expect, for the SR-2508 data in the RIF-1 tumour. Therefore, with the exception of this one case, we can say that combined with 50 mg kg$^{-1}$ CY each sensitizer enhances cytotoxicity significantly, but that the dependence on sensitizer concentration is unclear.

**References**

ARMITAGE, P. (1971). *Statistical Methods in Medical Research*. Oxford: Blackwell Sci. Publ., Section 13.3.

BROWN, D.M., GONZALES-MENDEZ, R. & BROWN, J.M. (1983). Factors influencing intracellular uptake and radiosensitization by 2-nitroimidazoles *in vitro*. *Radiat. Res.*, **93**, 492.

BROWN, J.M. (1983). Clinical trials of radiosensitizers: What should we expect? *Int. J. Radiat. Oncol. Biol. Phys.*, (in press).

BROWN, J.M. & HIRST, D.G. (1982). Effect of clinical levels of misonidazole on the response of tumour and normal tissues in the mouse to alkylating agents. *Br. J. Cancer*, **45**, 700.

BROWN, J.M., TWENTYMAN, P.R. & ZAMVIL, S.S. (1980). The response of the RIF-1 tumor *in vitro* and in $C_3H$/Km mice to X-radiation (cell survival, regrowth delay, and tumor control), chemotherapeutic agents, and activated macrophages. *J. Natl Cancer Inst.*, **64**, 605.

CONROY, P.J., McNEILL, T.H., PASSALACQUA, W., MERRITT, J., REICH, K.R. & WALKER, S. (1982). Nitroimidazole neurotoxicity: Are mouse studies predictive? *Int. J. Radiat. Oncol. Biol. Phys.*, **8**, 799.

HINCHCLIFFE, M., McNALLY, N.J. & STRATFORD, M.R.L. (1983). The effect of radiosensitizers on the pharmacokinetics of melphalan and cyclophosphamide in the mouse. *Br. J. Cancer*, **48**, 375.

HIRST, D.G. & BROWN, J.M. (1982). The therapeutic potential of misonidazole enhancement of alkylating agent cytotoxocity. *Int. J. Radiat. Oncol. Biol. Phys.*, **8**, 639.

HIRST, D.G., BROWN, J.M. & HAZLEHURST, J.L. (1983). Enhancement of CCNU cytotoxicity by misonidazole: Possible therapeutic gain. *Br. J. Cancer*, **46**, 109.

LAW, M.P., HIRST, D.G. & BROWN, J.M. (1981). The enhancing effect of misonidazole on the response of the RIF-1 tumour to cyclophosphamide. *Br. J. Cancer*, **44**, 208.

LEE, F.Y.F. & WORKMAN, P. (1983). Modification of CCNU pharmacokinetics by misonidazole – a major mechanism of chemosensitization in mice. *Br. J. Cancer*, **47**, 659.

LU, C.C. & MEISTRICH, M.L. (1979). Cytoxic effects of chemotherapeutic drugs on mouse testis cells. *Cancer Res.*, **39**, 3575.

McNALLY, N.J., HINCHCLIFFE, M. & DE RONDE, J. (1983). Enhancement of the action of alkylating agents by single high, or chronic low doses of misonidazole. *Br. J. Cancer*, **48**, 271.

SIEMANN, D.W. (1982). Response of murine tumors to combination of CCNU with misonidazole and other radiation sensitizers. *Br. J. Cancer*, **45**, 272.

TAYLOR, Y.C., EVANS, J.W. & BROWN, J.M. (1983). Mechanism of sensitization of Chinese hamster ovary cells to melphalan by hypoxic treatment with misonidazole. *Cancer Res.*, **43**, 3175.

TILL, J.E. & McCULLOCH, E.A. (1961). A direct measurement of the radiation sensitivity of normal mouse bone marrow cells. *Radiat. Res.*, **14**, 213.

TWENTYMAN, P.R., BROWN, J.M., GRAY, J.W., FRANKO, A.J., SCOLES, M.A. & KALLMAN, R.F. (1980). A new mouse tumor model system (RIF-1) for comparison of end-point studies. *J. Natl Cancer Inst.*, **64**, 595.

URTASUN, R.C., BAND, P., CHAPMAN, J.D., FELDSTEIN, M.L., MIELKE, B. & FRYER, C. (1976). Radiation and high-dose metronidazole in supratentorial glioblastomas. *N. Engl. J. Med.*, **294**, 1364.

WORKMAN, P. & BROWN, J.M. (1981). Structure-pharmacokinetic relationships for misonidazole analogues in mice. *Cancer Chem. Pharmacol.*, **6**, 39.

WORKMAN, P. & TWENTYMAN, P.R. (1982*a*). Structure-activity relationships for the enhancement by electron-affinic drugs of the anti-tumor effect of CCNU. *Br. J. Cancer*, **46**, 249.

WORKMAN, P. & TWENTYMAN, P.R. (1982*b*). Enhancement by electron-affinic agents of the therapeutic effects of cytotoxic agents against the KHT tumor: Structure activity relationships. *Int. J. Radiat. Oncol. Biol. Phys.*, **8**, 623.